# Application of Factor Analysis to Identify Dietary Patterns and Use of Factor Scores to Study Their Relationship with Nutritional Status of Adult Rural Populations

**DOI:** 10.3329/jhpn.v29i4.8448

**Published:** 2011-08

**Authors:** K. Venkaiah, G.N.V. Brahmam, K. Vijayaraghavan

**Affiliations:** Division of Community Studies, National Institute of Nutrition, Hyderabad, India

**Keywords:** Caloric intake, Chronic energy deficiency, Community-based studies, Cross-sectional studies, Discriminant function, Factor analysis, Nutritional status, Principal component analysis, India

## Abstract

The prevalence of chronic energy deficiency (CED) among one-third of the Indian population is attributed to inadequacy of consumption of nutrients. However, considering the complexity of diets among Indians, the relationship between a particular dietary pattern and the nutritional status of the population has not been established so far. A community-based cross-sectional study was undertaken to assess estimates, at district level, of diet and nutritional status in Orissa State, India. Factor analysis was used for exploring the existence of consumption pattern of food and nutrients and their relationship with the nutritional status of rural adult population. Data on 2,864 adult men and 3,525 adult women in Orissa state revealed that there exists six patterns among food-groups explaining 59% of the total variation and three patterns among nutrients that explain 73% of the total variation among both adult men and women. The discriminant function analysis revealed that, overall, 53% of the men were correctly classified as either with chronic energy deficiency (CED) or without CED. Similarly, overall, 54% of the women were correctly classified as either with CED or without CED. The sensitivity of the model was 65% for both men and women, and the specificity was 46% and 41% respectively for men and women. In the case of classification of overweight/obesity, the prediction of the model was about 75% among both men and women, along with high sensitivity. Using factor analysis, the dietary patterns were identified from the food and nutrient intake data. There exists a strong relationship between the dietary patterns and the nutritional status of rural adults. These results will help identify the community people with CED and help planners formulate nutritional interventions accordingly.

## INTRODUCTION

Thirty percent of rural adults in India currently suffer from chronic energy deficiency (CED). Ade-quate intakes of foods and nutrients are a major contributing factor for the maintenance of good health throughout the life. The complexity of human diets presents a challenge to those intending to study the prevalence of CED in relation to die-tary intakes. Food intake is generally studied in terms of adequacy of nutrients. However, foods contain other chemical compounds, some of which are established, some are poorly characterized, and others being completely unknown cannot be measured. In epidemiological point of view, the diet represents a complex set of highly-correlated exposures. Thus, the relationship between a food-group and the prevalence of CED may erroneously be attributed to a single component, undermining the fact that there exists multi-colinearity between nutrients and foods which can be demonstrated by employing sophisticated statistical procedure, such as factor analysis. In nutritional epidemiology, factor and cluster analyses are two commonly-used methods to derive eating patterns. Factor analysis reduces data into patterns based upon intercorrelations between dietary items whereas cluster analysis reduces data into patterns based upon individual differences in mean intakes. This approach uses the correlations between food and nutrient intake to describe general patterns that can be studied in relation to the nutritional status of people in the community.

Two statistical approaches have been used for developing general descriptions of dietary patterns. The first one is called ‘a priori’ which is based on previous knowledge of necessary nutrients to maintain the needs of the body. Another approach is a ‘posteriori’, which is based on the actual intakes. The main techniques in the latter approach are principal component analysis (PCA), followed by factor analysis. The objective is to transform a large set of correlated variables into smaller sets of non-correlated variables, called principal components or factors (1). In factor analysis, rather than establishing a diet indicator, data objectively point towards how measurements are clustered. The aim of this technique is to identify the underlying structure in data matrix, by summarizing and consigning data to arrive at a systematic measurement of the diet. To summarize data, factor analysis desires dimension that, when interpreted and understood, describes data in terms of a much smaller number of items than do the individual variables.

The objective of the present analysis was to describe the food and nutrient-consumption patterns in the rural community in Orissa state, India, which are apparently homogeneous and to relate these with the prevalence of CED and overweight/obesity.

## MATERIALS AND METHODS

### Study design and sample

The study used data from a district nutrition profile survey in Orissa state carried out with the support of the Department of Women and Child Development, Government of India. In total, 12,000 households—400 households per district—from 30 districts in Orissa state were covered (2). Anthropometric measurements, viz. height and weight, were taken on all the available individuals in the households. In every alternate household covered for nutritional assessment, a family diet survey was carried out by 24-hour recall method. Individual dietary intakes were also assessed on 12,621 individuals of different age and gender-groups. From the average daily intake of foods, nutrients were computed using food-composition tables (3). The present analysis included data on 2,864 adult men and 3,525 adult women, aged ≥18 to <80 years, who were covered for the anthropometry and diet survey.

### Statistical analysis

The mean, median, and inter-quartile range of various food-groups and nutrients for men and women were calculated. Diet patterns were obtained by exploratory factor analysis for 13 food-groups and 11 (3 macro and 8 micro) nutrients. The nutrient composition of foods was estimated using the food-composition tables (3). Factor analysis—a multivariate statistical technique—was used for the identification of factors in a set of measurements. Such factors would correspond to indicators, and all variables were considered simultaneously, each one in relation to the others. For the applicability of factor analysis, the uniformity of sample was tested by examining the distribution of variables in a loading plot, contrasting the value observed against those expected in a normal distribution which was verified by Kaiser-Meyer-Olkin (KMO) measurement of adequacy. A KMO value of more than 0.50 was considered acceptable. The presence of correlations between food/nutrient-groups was tested using the Bartlett test of sphericity (homogeneity of variance). The Bartlett test statistic is approximately distributed with chi-square and was accepted when it is significant at p<0.05. PCA was used for extraction of factors and orthogonal rotation (varimax option) to derive non-correlated factors (4). This varimax method attempts to minimize the number of indicators that have high loading on one factor (5). The first factor extracted is the one that accounts for the maximum possible variance in the dataset. The second component, independent of the first, will be the one that explains the largest possible share of the remaining variance and so on, without the components being correlated with each other (6).

Kaiser criterion, namely Eigen value of >1.0, is the widely-used criterion for the choice of the number of factors in factor analysis. It was also based on the Eigen plot (scree plot), which shows the total variance associated with each other.

Body mass index (BMI) has been computed using height and weight of a given individual (weight in kg/height in metre^2^)*100 (7). The adult men and women studied were categorized into one of the two groups, i.e. with CED (BMI <18.5) as Group 1 and without CED (BMI ≥18.5) as Group 2 using James classification (8).

To study the prevalence of overweight/obesity, Asian cut-off points were used (9). The subjects with the BMI of ≥23.0 were classified as overweight/obese, and the remaining ones were treated as normal subjects.

Discriminant function analysis was carried out using the food and nutrient factor scores derived through the factor analysis to determine as to how correctly those people who were chronic energy-deficient or overweight/obese are classified. Statistical analysis was performed using the SPSS software (version 15.0).

### Ethical approval

The study was approved by the institutional ethical committee of the National Institute of Nutrition, and oral informed consent was obtained from the village heads.

**Table 1.** Average intake (g/day) of foods among rural adult population by gender (men−2,864 and women−3,525)

Food-group	Gender	Mean	Median	SD	25th and 75th percentiles
Cereals and millets	Men	660.1	642.5	246	502.7-802.0
	Women	534.5	520.5	200	400.0-650.2
Pulses and legumes	Men	36.5	27.8	44.8	0.0-55.6
	Women	29.9	21.1	38.3	0.0-45.0
Green-leafy vegetables	Men	37.7	0.0	72.9	0.0-55.6
	Women	31.8	0.0	59.4	0.0-47.6
Roots and tubers	Men	108.4	84.8	106.0	31.3-152.5
	Women	85.1	70.8	76.5	25.0-125.0
Other vegetables	Men	106.0	80.0	109.0	19.1-154.1
	Women	82.6	63.5	83.6	13.8-120.0
Nuts and oilseeds	Men	1.2	0.0	6.9	0.0-0.0
	Women	0.9	0.0	4.9	0.0-0.0
Condiments and spices	Men	7.6	5.2	9.9	2.7-9.2
	Women	6.3	4.2	8.7	2.1-7.5
Fruits	Men	18.7	0.0	37.1	0.0-22.5
	Women	13.8	0.0	29.4	0.0-15.7
Fish and other sea-foods	Men	13.6	0.0	39.6	0.0-0.0
	Women	10.6	0.0	32.3	0.0-0.0
Meat and poultry	Men	3.7	0.0	25.4	0.0-0.0
	Women	2.8	0.0	20.0	0.0-0.0
Milk and milk products	Men	23.3	0.0	54.4	0.0-28.4
	Women	17.9	0.0	44.6	0.0-12.2
Fats and oils	Men	9.3	7.0	10.3	4.0-11.5
	Women	7.4	5.3	8.2	3.1-9.3
Sugar and jaggery	Men	14.2	7.0	21.9	0.0-20.0
	Women	12.1	5.0	21.9	0.0-16.8

SD=Standard deviation

**Table 2.** Average intake (g/day) of nutrients among rural adult population by gender (men−2,864 and women−3,525)

Nutrient	Gender	Mean	Median	SD	25th and 75th percentiles
Protein (g)	Men	63.2	60.1	24.3	47.5-76.0
	Women	51.0	48.4	19.8	37.7-61.2
Total fat (g)	Men	16.4	13.1	12.9	9.2-20.0
	Women	13.1	10.4	10.2	7.1-15.9
Energy (Kcal)	Men	2,762	2,688	931.5	2,148-3,277
	Women	2,233	2,169	756.0	1,725-2,641
Calcium (mg)	Men	488	333	657.6	187.9-601.0
	Women	398	268	516.6	153.8-482.9
Iron (mg)	Men	16.7	13.6	13.4	10.0-18.9
	Women	13.6	10.9	10.7	8.1-15.5
Vitamin A (µg)	Men	350.7	73.1	718.0	37.7-183.3
	Women	301.6	60.8	611.8	29.6-160.6
Thiamine (mg)	Men	1.8	1.7	0.7	1.3-2.2
	Women	1.4	1.4	0.6	1.1-1.8
Riboflavin (mg)	Men	0.65	0.6	0.3	0.5-0.8
	Women	0.53	0.5	0.2	0.4-0.6
Niacin (mg)	Men	27.2	26.7	10.3	20.3-33.3
	Women	22.0	21.7	8.4	16.3-27.0
Vitamin C (mg)	Men	83.4	47.8	100.3	24.7-104.3
	Women	66.9	39.0	78.8	20.0-83.5
Free Folic acid (µg)	Men	166.5	142.2	100.2	97.5-206.9
	Women	136.2	116.7	81.9	80.4-169.4

SD=Standard deviation

## RESULTS

The average and interquartile range of food and nutrients intake according to gender is presented in Table 1 and 2. A wide variation in the intake of foods and nutrients was observed in both male and female adult populations. The average intake of iron, calcium, and riboflavin was lower in both men and women of the present study compared to the re-commended dietary allowances (10). The study by Venkaiah *et al.* among the Indian rural adolescents observed that the average intakes of all the nutrients (11) were below the recommended dietary allowances. The nutrients were determined using the food-composition tables extensively used in India. The nutrients for each food item consumed for the day were calculated, and the nutrients were pooled. The food grouping was done as is adopted in the National Nutrition Monitoring Bureau surveys in India over the past 38 years and is well-accepted by nutritionists. In the context of undernutrition in developing countries, particularly India, energy is the major bottleneck in dietaries contributing to extensive protein-energy malnutrition. Therefore, nutritionists consider energy as an important nutrient arriving at the energy value of a food item. Energy is calculated from the analysis of foods for protein, fat, carbohydrate, and multiplication of the content of these components with appropriate energy value of these factors. PCA has been done considering the methods of presentation and interpretation of data by the nutritionists.

Free folic acid refers to the biologically-active form of folic acid, since all the total folic acid available in the diet is itself not biologically active.

The adequacy of data was evaluated based on the value of KMO and (homogeneity of variance). The KMO measure compares the value of partial correlation coefficients against the total correlation coefficients. The maximum value of this measure is 1, and the larger value indicates that most partial correlations are small compared to the total correlations. A KMO value of 0.90 is considered marvelous, 0.80 meritorious, 0.70 middling, 0.60 mediocre, and 0.50 miserable. This measure also represents the adequacy of sample-size of foods among men and women, which was 0.543 and 0.544 respectively, indicating that sufficient correlation existed between different foods to proceed with factor analysis. Similarly, in the case of nutrients, the KMO measure for men and women was 0.741 and 0.761, indicating a higher correlation between nutrients compared to that between foods. The Bartlett test of sphericity for foods and nutrients among males and females was highly significant (p<0.001), indicating homogeneity of variance by the consumption of foods and nutrients.

Table 3 reveals that six components each for both male and female populations were extracted by factor analysis using PCA with varimax rotation for food-groups. The first six components (factors) in the initial solution have an Eigen value over 1, and they account for about 59% of the observed variation in the food-consumption pattern among the adult males. Similarly, even among the women, the first six factors accounted for about 60% of the observed variation in the food-consumption pattern (Table 3). According to Kaiser criterion having Eigen value of >1 only should be considered for interpretation. The parallel line to horizontal at Eigen value equalling to 1 in scree plot showed that six factors will be extracted for foods in both male and female population (Fig.1a and Fig.1b).

**Table 3.** Rotated component matrix for food-groups

Food-group (g)	Adult men component						Adult women component					
	1	2	3	4	5	6	1	2	3	4	5	6
Cereals and millets	-0.32	0.36	0.35	0.35	-0.26	-0.04	-0.28	0.19	0.49	-0.16	0.38	0.04
Pulses and legumes	0.33	0.14	0.54	-0.11	-0.41	0.00	0.38	0.02	0.62	-0.23	-0.03	-0.08
Green-leafy vegetables	0.04	-0.24	-0.10	0.77	0.00	0.10	0.01	-0.22	-0.19	-0.03	0.78	0.06
Roots and tubers	0.05	0.72	-0.01	0.06	0.21	0.06	0.05	0.74	-0.12	0.21	0.06	0.05
Other vegetables	0.13	0.69	-0.06	-0.04	-0.22	-0.05	0.06	0.70	0.12	-0.29	-0.08	-0.06
Nuts and oilseeds	0.18	-0.15	0.30	0.04	0.11	-0.67	0.15	-0.11	0.28	0.17	-0.06	-0.53
Condiments and spices	-0.15	-0.09	0.69	-0.02	0.19	0.02	-0.17	-0.06	0.56	0.36	-0.03	0.08
Fruits	0.02	0.26	0.04	0.66	0.01	-0.09	0.07	0.17	0.18	0.05	0.57	-0.08
Fish and other sea-foods	-0.02	0.02	0.08	-0.04	0.81	-0.08	-0.02	-0.01	-0.05	0.80	-0.02	-0.10
Meat and poultry	0.15	-0.09	0.28	0.03	0.02	0.71	0.14	-0.06	0.20	0.07	-0.08	0.83
Milk and milk products	0.74	0.14	-0.09	-0.05	0.06	0.07	0.72	0.15	-0.11	0.03	-0.06	0.08
Fats and oils	0.32	0.43	0.34	0.07	0.39	0.21	0.32	0.45	0.23	0.46	0.11	0.14
Sugar and jaggery	0.80	0.03	0.05	0.10	-0.10	-0.08	0.80	-0.01	0.09	-0.02	0.10	-0.05
Eigen value	1.94	1.35	1.22	1.09	1.09	1.02	1.87	1.28	1.21	1.13	1.03	1.03
Variance explained	14.96	10.39	9.38	8.40	8.38	7.84	14.41	9.84	9.29	8.70	7.95	7.79

Extraction method;

Principal component analysis;

Rotation method;

Varimax with Kaiser normalization;

Rotation converged in 7 iterations

The first factor, which accounted for 12.2% of the total variance among the males was labelled as income-elastic foods. High factor loading observed for milk and milk products and sugar and jaggery characterized these factors. The second factor explained 11.6% of the total variance and was labelled as plant-foods (roots and tubers and other vegetables). The third factor accounted for 9.3% of the total variance, and this factor was characterized by the intake of pulse and legumes, and condiments and spices and was labelled as traditional. The fourth factor explained 9.2% of the total variance and labelled as micronutrient-rich foods, such as green-leafy vegetables and fruits. The fifth factor accounted for 9.2% of the total variance and characterized by the intake of fish and sea-foods, and it was labelled as aquatic foods. The last and the sixth factor explained 8.0% of the total variance and was labelled as protein-rich foods (nuts and oilseeds, and meat and poultry).

**Table 4.** Rotated component matrix for nutrients

Nutrient	Adult men component			Adult women component		
	1	2	3	1	2	3
Proteins (g)	0.82	0.03	0.43	0.82	0.03	0.44
Total fat (g)	0.28	0.10	0.55	0.25	0.06	0.60
Energy (Kcal)	0.93	0.01	0.18	0.94	0.01	0.19
Calcium (mg)	-0.03	0.16	0.84	-0.04	0.17	0.83
Iron (mg)	0.22	-0.02	0.66	0.31	0.01	0.56
Vitamin A (μg)	0.06	0.90	0.12	0.02	0.90	0.12
Thiamine (mg)	0.92	0.07	0.16	0.93	0.05	0.17
Riboflavin (mg)	0.72	0.42	0.26	0.70	0.42	0.29
Niacin (mg)	0.92	0.06	0.03	0.93	0.05	0.04
Vitamin C (mg)	0.11	0.85	0.05	0.10	0.85	0.05
Free folic acid (μg)	0.64	0.46	0.12	0.62	0.46	0.13
Eigen value	5.17	1.66	1.24	5.16	1.73	1.14
Variance explained	47.00	15.10	11.20	46.87	15.76	10.35

Extraction method;

Principal component analysis;

Rotation method;

Varimax with Kaiser normalization;

Rotation converged in 5 iterations

Interestingly, the data indicate similar factor loadings, and approximately the same percentage of total variance was explained by each of the factors in food consumption by adult women as well (Table 3).

Table 4 revealed that, in the case of nutrients, three components each for both male and female population were extracted by factor analysis using PCA with varimax rotation. It was observed that the variables, such as energy, thiamine, niacin, protein, riboflavin, and free folic acid, had loadings of 0.934, 0.924, 0.923, 0.817, 0.722, and 0.639 respectively on factor 1 among the men. This factor explained 39.3% of the total variance and was labelled as macronutrients and B-complex vitamins. Factor 2 labelled as ‘vitamins’ contains vitamin A and C and had a high loading of 0.896 and 0.850, which explains 17.8% of the total variation. The third factor accounted for 16.3 % of the total variance. Since this factor was characterized by the intake of total fat, calcium, and iron, it was labelled as fats and minerals. The parallel line to horizontal at Eigen value equalling to 1 in scree plot showed that the three factors will be extracted for nutrients in both male and female population (Fig. 1c and 1d).

The data indicate similar factor loadings, and approximately the same percentage of total variance was explained by each of the factors in consumption of nutrients also by the adult women (Table 4).

Figure 2a and 2b show the graphical spatial representation of derived factors for foods and Figure 2c and 2d for nutrients among men and women separately. In this graph, the groupings of variables and their relationship with the derived factors can be seen.

**Fig. 1a.**
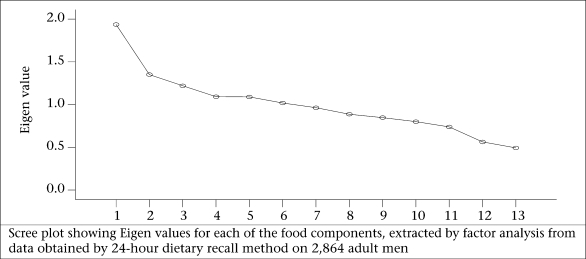
Intake of food-groups among men

**Fig. 1b.**
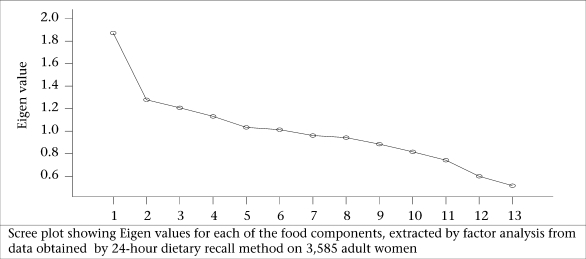
Intake of food-groups among women

**Fig. 1c.**
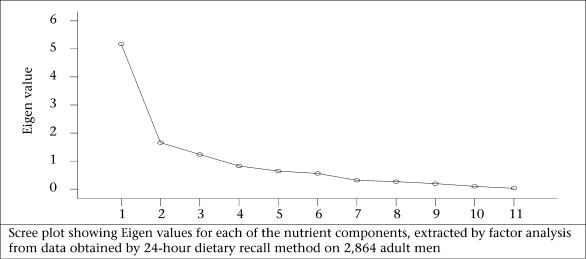
Intake of nutrients among men

**Fig. 1d.**
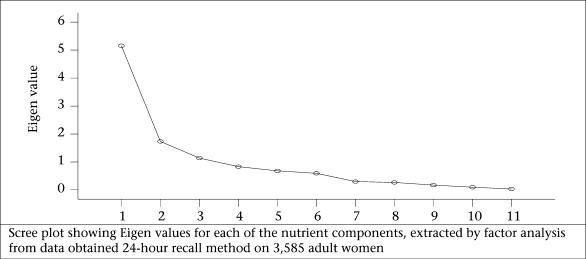
Intake of nutrients among women

**Fig. 2a.**
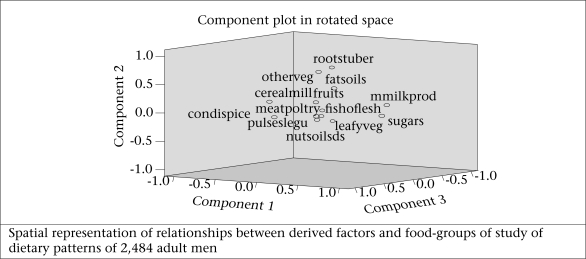
Intake of food-groups among men

**Fig. 2b.**
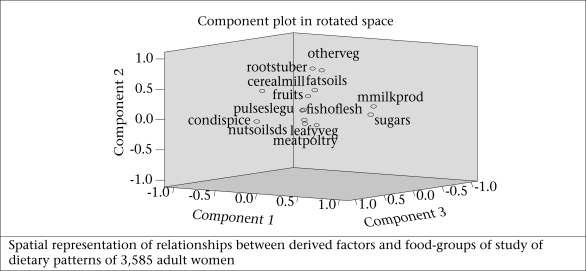
Intake of food-groups among women

**Fig. 2c.**
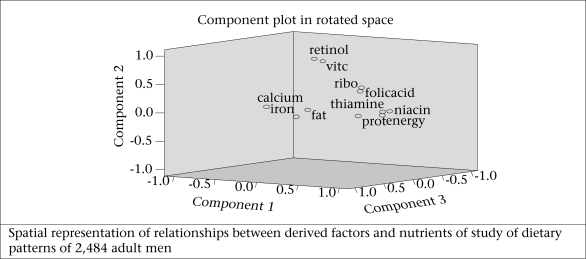
Intake of nutrients amon g men

**Fig. 2d.**
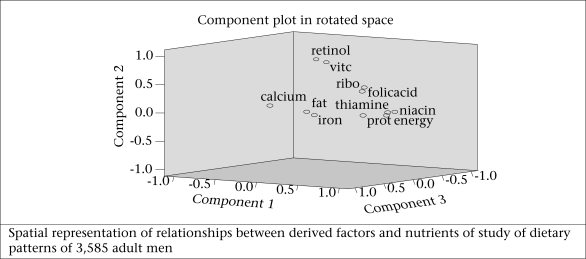
Intake of nutrients among women

Discriminant function analysis was used for studying the relationship between the food and nutrient intake and the nutritional status. The factor scores obtained by the factor analysis for food and nutrients were considered continuous independent variables, and BMI category of the individuals was considered dichotomous dependent variable.

The prevalence of CED (BMI <18.5) was 37.5% among the men and 55% among the women while the prevalence of overweight/obesity (by using Asian cut-off point 23) was 3.2% and 2.9% among men and women respectively.

The distributions of adults predicted to have CED based on the derived BMI values by the discriminant function analysis using the factor scores obtained from factor analysis for various foods and nutrients as against those based on the observed CED are presented in Table 5a and 5b. Based on the discriminant function analysis, overall, 53% of the men with and without CED were predicted correctly. In other words, the sensitivity of discriminanat function was 65%, and the specificity was 46%. Similarly, 54.2% of the women with and without CED were predicted correctly, with a sensitivity of 65% and a specificity of 41%.

Similar distribution based on the predicted number of adult men with overweight/obesity using the discriminate function analysis indicated that 74% of those observed to have overweight/obesity and not having overweight/obesity were classified correctly by the discriminant function, with a sensitivity of 75% and a specificity of 49%. In the case of women, 74.9% were classified correctly, with a sensitivity of 65% and a specificity of 41% (Table 6a and 6b).

**Table 5.** Classification of subjects with CED and without CED using observed BMI and predicted BMI using scores derived from factor analysis for foods and nutrients

a. Classification results: men					
	BMI		Predicted BMI group according to factor scores derived through factor analysis		Total
			<18.5	≥18.5	
According to BMI	Count	<18.5	700	374	1,074
		≥18.5	971	819	1,790
	%	<18.5	65.2	34.8	100
		≥18.5	54.2	45.8	100
Sensitivity [(test positives (700)/actual positives (1,074)] is 65%, and specificity [(test negatives (819)/actual negatives (1,790)] is 46%					
b. Classification results: women					
	BMI		Predicted BMI group according to factor scores derived through factor analysis		Total
			<18.5	≥18.5	
According to BMI	Count	<18.5	1,265	678	1,943
		≥18.5	938	644	1,582
	%	<18.5	65.1	34.9	100
		≥18.5	59.3	40.7	100
Sensitivity [(test positives (1,265)/actual positives (1,943)] is 65%, and specificity [(test negatives (644)/actual negatives (1,582)] is 41%					

BMI=Body mass index; CED=Chronic energy deficiency

**Table 6.** Classification of subjects with and without overweight/obesity using observed BMI and predicted BMI using scores derived from factor analysis for foods and nutrients

a. Classification results: men					
	BMI		Predicted BMI group according to factor scores derived through factor analysis		Total
			<23	≥23	
According to BMI	Count	<23	2,074	698	2,772
		≥23	47	45	92
	%	<23	74.8	25.2	100
		≥23	51.1	48.9	100
Sensitivity [(test positives (2,074)/actual positives (2,772)] is 75%, and specificity [(test negatives (45)/actual negatives (92)] is 49%					
b. Classification results: women					
	BMI		Predicted BMI group according to factor scores derived through factor analysis		Total
			<23	≥235	
According to BMI	Count	<23	2,595	828	3,423
		≥23	55	47	102
	%	<23	65.1	34.9	100
		≥23	59.3	40.7	100
Sensitivity [(test positives (2,595)/actual positives (3,423)] is 65%, and specificity [(test negatives (47)/actual negatives (102)] is 41%					

BMI=Body mass index

## DISCUSSION

Analysis of dietary patterns may offer benefits of summarizing diet using a small number of factors. Yet, these may be potentially of greater relevance to educate the general public on more healthful eating habits. Development of robust and meaningful techniques of dietary pattern analysis is useful to understand the role of dietary patterns in health and disease. Results of a study revealed that both PCA and cluster analysis are useful approaches for the assessment of dietary patterns (12). A commonly-cited criticism of the two techniques is that these involve several subjective—but important—decisions, such as grouping of foods and nutrients and possible transformations of variables. PCA involves decisions about the number of components to be retained and their subsequent labelling. Similarly, cluster analysis requires choices about the method of clustering and labelling of clusters. Another disadvantage of these techniques is that they generate patterns based on variation in diet, with no guarantee that these patterns will be predictive of a particular health outcome. However, the techniques have the advantage that they are empirically derived and are, therefore, not limited by a mere priori knowledge.

In developing countries, studies on identifying dietary patterns and their relationship with nutritional status are scarce. The present analysis explores the possible dietary patterns among the adult rural population using factor analysis. Six patterns among foods explaining 59% of the total variability and three patterns among nutrients explaining 73% of the total variation were identified.

In foods, the first factor was characterized by the presence of income-elastic foods, such as milk and milk products, and sugar and jaggery. The impact of the intake of milk and milk products on health seems to be important and indirectly reflects the socioeconomic status of the households. The majority of the rural population consumes roots and tubers and other vegetables which were characterized as second factor and labelled as plant-foods. An equal contribution of other factors (third, fourth, and fifth) was identified by the analysis. The sixth factor labelled as protein-rich foods contributed about 8% of the variation in men and in women.

The first factor among nutrients was characterized by the presence of macronutrients and B complex vitamins, and their contribution to nutrition status seems to be very important. The second factor was characterized by the presence of vitamins, such as vitamin A and vitamin C, which are important for maintaining good health. The third pattern consists of total fat, calcium, and iron which are necessary for the body.

The intake of foods and nutrients highly correlated, and the classification methods based on univariate analysis may lead to flawed estimates. Therefore, multivariate methods, such as factor analysis, represent an alternative approach to the evaluation of individual foods and nutrients since the identification of patterns allows us to examine the effect of diet as a whole and helps describe the association with nutritional status. Moreover, it should be kept in mind that individuals consume nutrients based on their food-choices, which are influenced by many factors, such as cultural, socioeconomic and demographic characteristics. Describing food intake in different consumption patterns may be useful in developing community programmes. Rather than changing the nutrient intake, such programmes can be aimed at changing the intake of foods that are readily recognized by the target group. It is possible to find a smaller number of measures using factor analysis that would permit the identification of persons who are nutritionally at risk (13).

The factor scores obtained for men and women separately through factor analysis were used for the prediction and classification of adults having CED based on BMI values. Newby *et al*. (2004) in their review reported that, although several studies examined the relationship between dietary patterns and nutritional status, overweight, obesity, etc., there were many consistencies in establishing a clear relationship between them (14). However, the present data showed a considerable relationship between the dietary pattern and the nutritional status. About 65% of men and women with CED were correctly classified as chronic-energy deficient (sensitivity), and 75% of men and 65% of women were correctly classified as overweight/obese based on scores obtained by factor analysis.

The dietary patterns that are extracted from the data obtained in the study may not be applicable to other populations with different food habits.

### Conclusions

The data allowed the identification of dietary patterns defined by factor analysis based on the data from food and nutrient intake. There exists a strong relationship between specific diet pattern and CED and overweight/obesity among the rural population.

## ACKNOWLEDGEMENTS

The authors thank Dr. B. Sesikeran, Director, National Institute of Nutrition, for permission to conduct the study. They also thank Dr. M. Vishnuvardhan Rao and Dr. N. Balakrishna for their technical support.
